# A Rare Case of Sarcoidosis With Aggressive Bony Lesion in the Frontal Bone

**DOI:** 10.7759/cureus.99316

**Published:** 2025-12-15

**Authors:** Abhishek Bhattacharjee, Asma Khanom

**Affiliations:** 1 Acute Medicine, Peterborough City Hospital, Peterborough, GBR

**Keywords:** corticosteroid therapy, cranial neuropathy, frontal bone lesion, granulomatous disease, multidisciplinary management, non-caseating granuloma, osseous sarcoidosis, sarcoidosis, serum ace level, skull sarcoidosis

## Abstract

Sarcoidosis is a multisystem granulomatous disorder that can rarely involve bone, particularly the skull, where lesions may closely resemble malignant or infectious processes. We report a 60-year-old man with established multisystem sarcoidosis who presented with acute left-sided headache, diplopia, and periorbital pain. Neuroimaging demonstrated a poorly defined left frontal bone lesion with homogeneous post-contrast enhancement. Laboratory evaluation revealed elevated angiotensin-converting enzyme levels, and a supraclavicular lymph-node biopsy confirmed non-caseating granulomas. As systemic sarcoidosis had already been histologically established and the radiologic features were characteristic, direct biopsy of the cranial lesion was deferred. The patient was treated with oral corticosteroids, resulting in rapid improvement in pain and cranial-nerve function. This case highlights the importance of recognising cranial osseous sarcoidosis as a potential mimic of malignancy to avoid unnecessary invasive procedures and emphasises the value of timely corticosteroid therapy and coordinated multidisciplinary management.

## Introduction

Sarcoidosis is a multisystem inflammatory granulomatous disorder of uncertain aetiology, histologically characterised by non-caseating granulomas that may involve one or more organ systems, most frequently the lungs, skin, eyes, and lymph nodes [1-3]. Pulmonary involvement occurs in nearly 90% of patients, while constitutional symptoms such as fatigue, fever, and weight loss are also common [2,3]. The clinical course is highly variable, ranging from spontaneous remission to chronic progressive multisystem disease [3]. Osseous sarcoidosis is relatively uncommon, reported in only 1-13% of cases, and typically affects the small bones of the hands and feet [1,3]. Cranial skeletal involvement is particularly uncommon [1,3].

Epidemiological studies show significant racial variation in sarcoidosis incidence. African-American women have the highest age-adjusted incidence, reaching approximately 39 cases per 100,000, followed by African-American men (~30 per 100,000) and Caucasian women (~12 per 100,000). Caucasian men have the lowest reported incidence (~10 per 100,000). African-American women aged 30-39 years are at the greatest risk, with incidence rates exceeding 100 per 100,000. Overall, the African American population has nearly a threefold higher incidence compared with the Caucasian population [4].

The disorder results from dysregulated T-cell-mediated immune activity against unidentified antigens in genetically predisposed individuals [5,6]. Although spontaneous remission is common, a proportion of patients develop chronic, multisystem, or progressive disease that necessitates immunosuppressive therapy [3,7]. Mortality in sarcoidosis is generally low, with reported rates ranging from approximately 1% to 6%, depending on the extent and severity of organ involvement [3].

Here, we report an unusual case of frontal-bone sarcoidosis presenting with features mimicking metastatic or neoplastic pathology, illustrating the diagnostic complexity and the importance of multidisciplinary evaluation.

## Case presentation

A 60-year-old man of African descent presented to the Ambulatory Care Unit with a three-day history of severe left-sided headache, eye pain, and diplopia. The headache was throbbing (10/10), localized over the left forehead and orbit, and associated with nausea but no vomiting or photophobia. He denied fever, weakness, or visual loss.

He had a prior diagnosis of sarcoidosis (skin, ocular, and lymphoreticular involvement) confirmed by endobronchial ultrasound-guided transbronchial needle aspiration and skin biopsy in 2017, managed conservatively without corticosteroids. His comorbidities included type 2 diabetes and previously treated latent tuberculosis (six-month regimen completed in 2018). Neurological examination revealed an isolated left sixth-cranial-nerve palsy without papilledema or other focal deficits. Laboratory evaluation showed an elevated serum angiotensin-converting enzyme (ACE) level of 75 U/L (normal 20-70 U/L), while calcium and alkaline phosphatase levels were normal.

**Figure 1:**
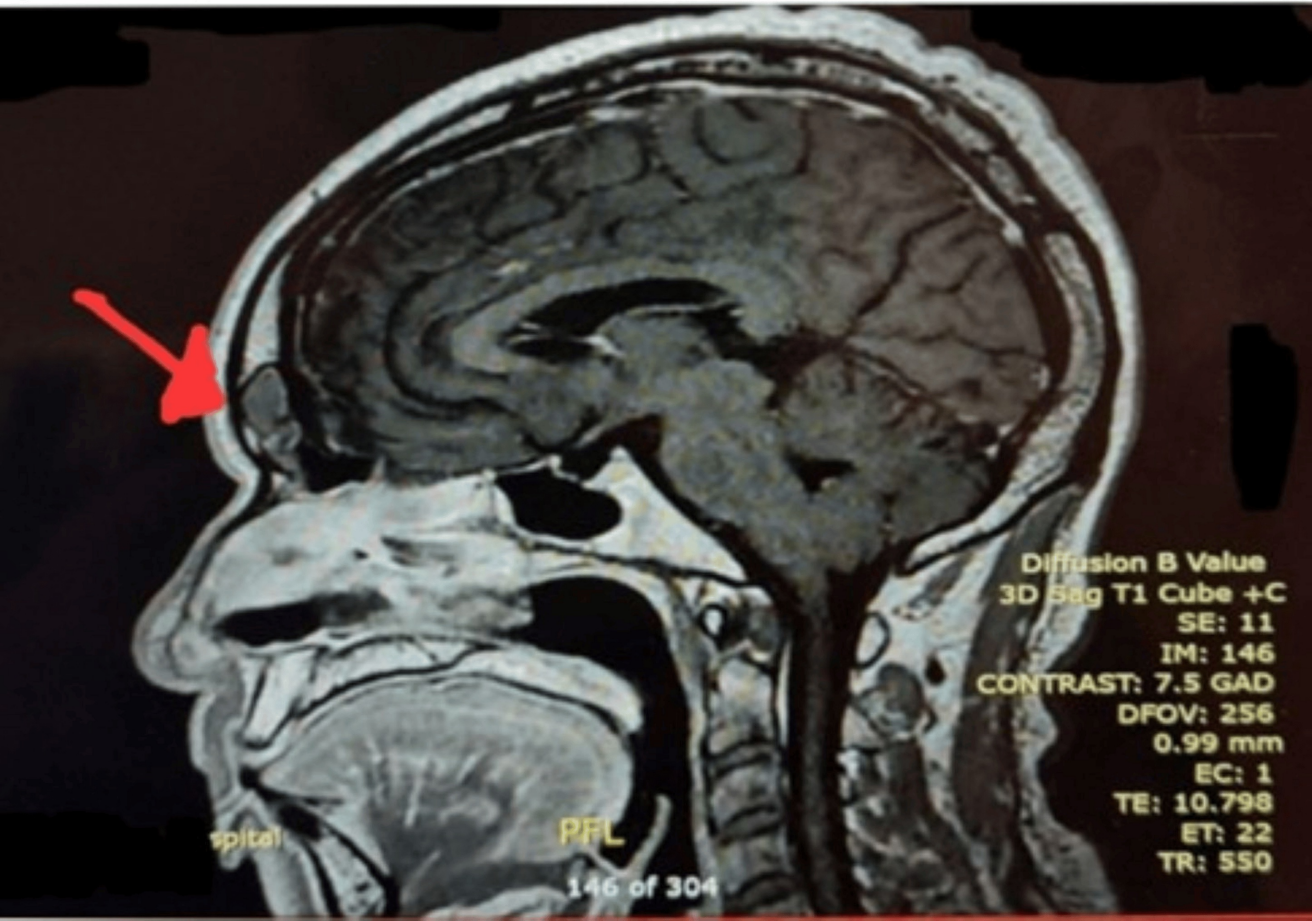
MRI brain (T1-weighted, sagittal view, post-contrast) showing a poorly defined calvarial lesion in the left frontal bone measuring approximately 34 mm with low signal intensity on T1-weighted images (arrow).

**Figure 2:**
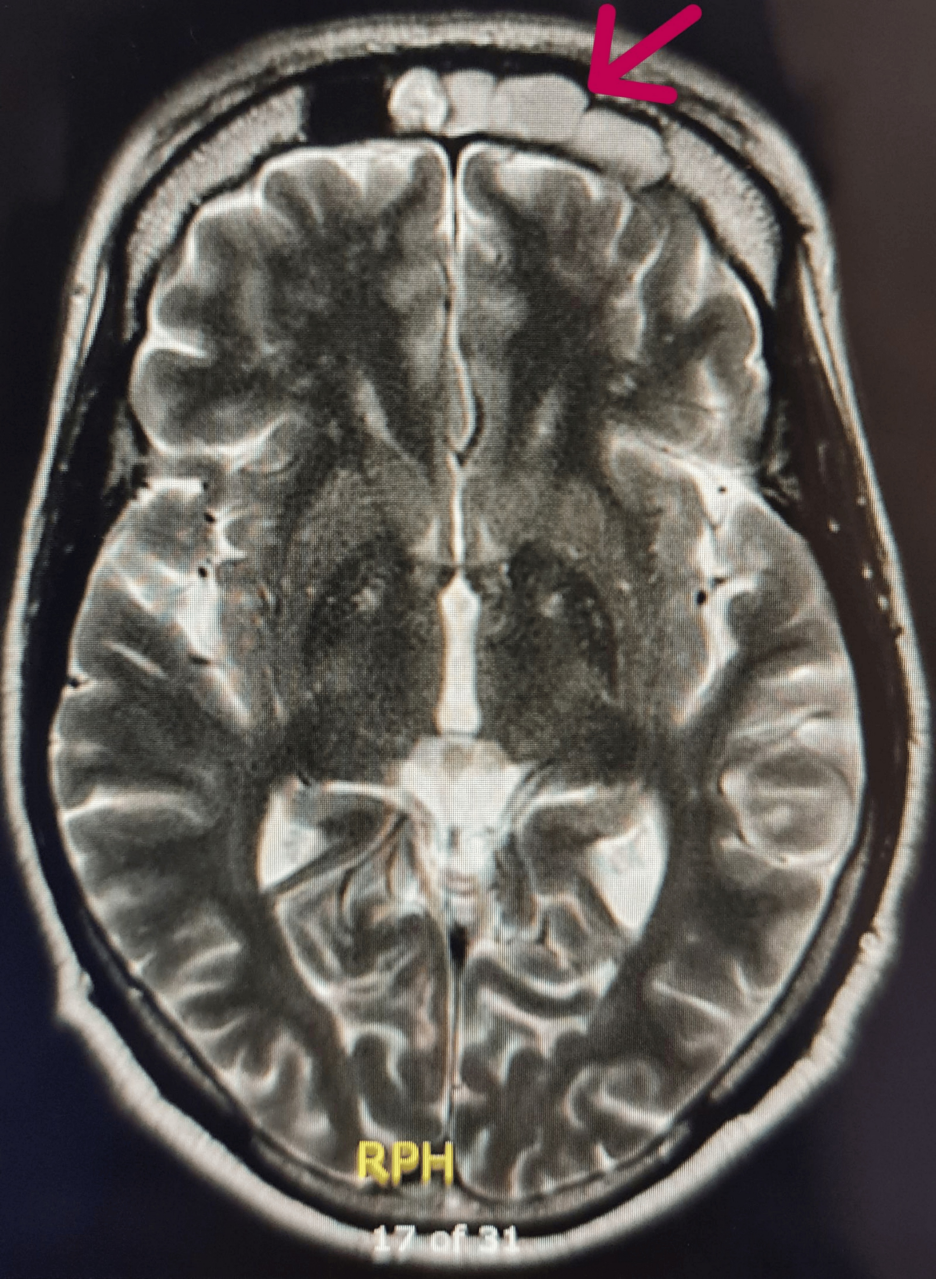
MRI brain (T2-weighted, axial view) showing intermediate signal intensity in the same left frontal bone lesion (arrow).

Magnetic resonance imaging (MRI) of the brain demonstrated a poorly defined 34-mm lesion in the left frontal bone with low T1 and intermediate T2 signal intensity and homogeneous post-contrast enhancement (Figures 1, 2).

**Figure 3:**
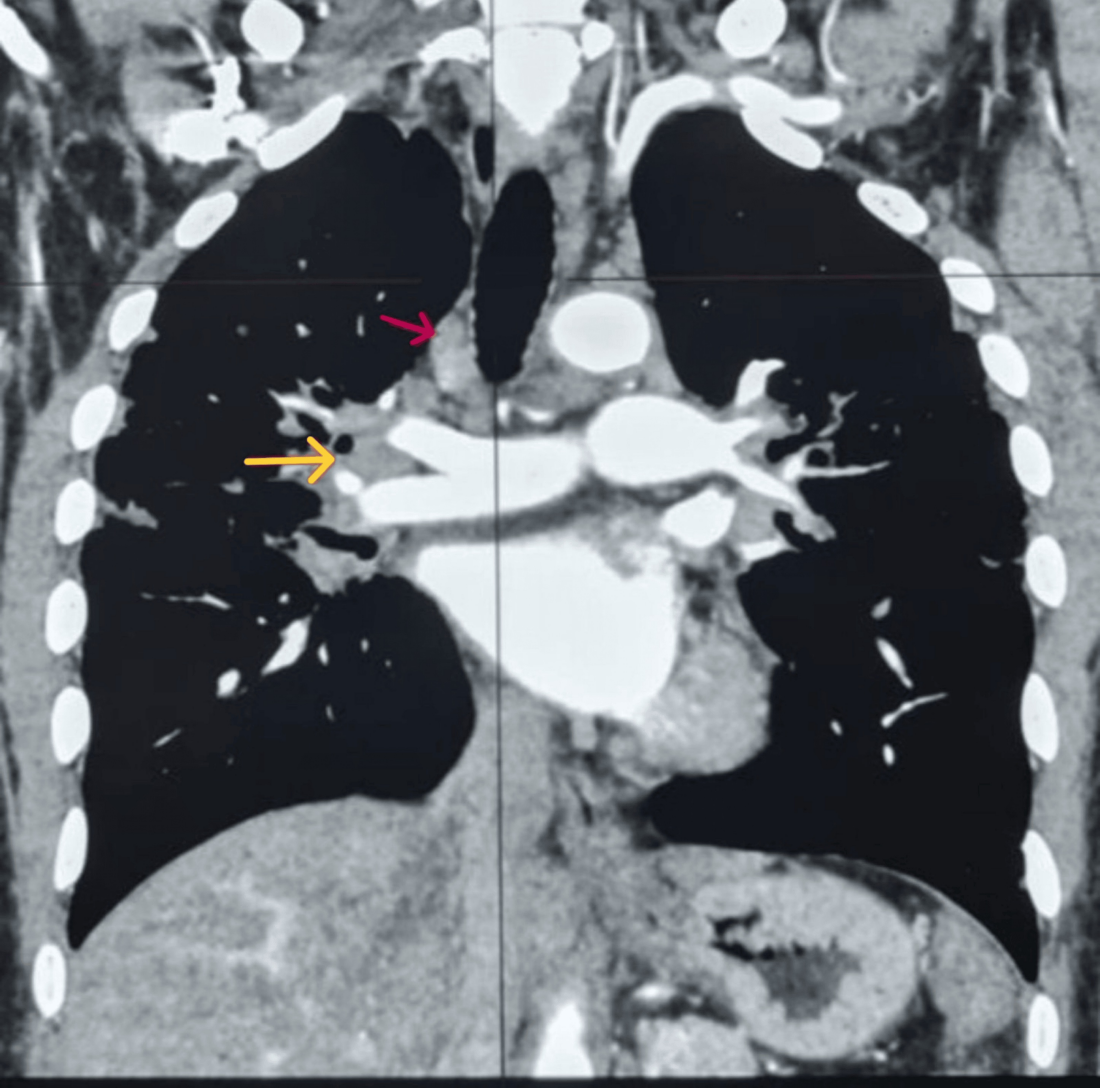
CT chest (coronal reconstruction, mediastinal window, contrast-enhanced) demonstrating anterior superior mediastinal lymphadenopathy (red arrow) and right hilar lymphadenopathy (yellow arrow).

**Figure 4:**
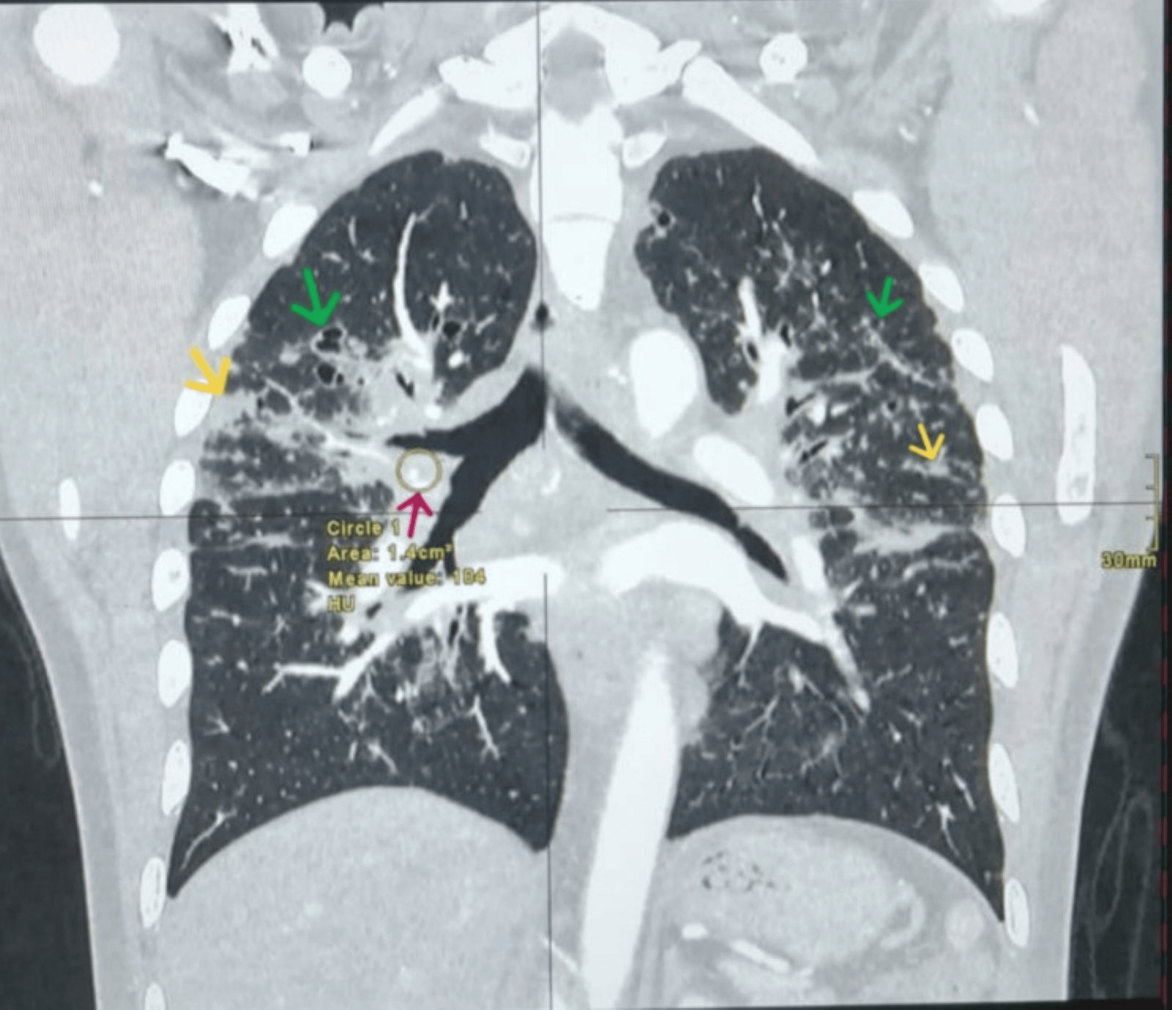
CT chest (coronal reconstruction, lung window, contrast-enhanced) demonstrating right hilar lymphadenopathy (red arrow), multiple bilateral subpleural nodules (yellow arrows), and cavitating pulmonary changes (green arrows).

The lesion extended slightly beyond the outer cortex with a small epidural component but no mass effect or intracranial involvement. Computed tomography (CT) of the chest, abdomen, and pelvis revealed supraclavicular, hilar, and mediastinal lymphadenopathy, subpleural nodules, and bilateral cavitating pulmonary changes (Figures 3, 4). 

Excision biopsy of the left supraclavicular lymph node showed florid, compact, non-caseating granulomas without evidence of malignancy, consistent with sarcoidosis. Cerebrospinal fluid analysis was normal. The neurology team attributed the sixth nerve palsy to microvascular ischemia secondary to diabetes rather than neurosarcoidosis, as there was no radiological evidence of meningeal disease, the cerebrospinal fluid was normal, and the palsy resolved spontaneously over time. While the sixth nerve palsy was attributed to diabetic microvascular disease, a minor, radiologically occult inflammatory component from neurosarcoidosis cannot be entirely excluded, and its resolution may have been aided by corticosteroid therapy.

Rationale for not performing a bone biopsy

A direct biopsy of the frontal bone lesion was not performed because systemic sarcoidosis had already been histologically confirmed from the lymph node. The imaging findings, presence of concurrent pulmonary and nodal disease, and subsequent rapid improvement with corticosteroid therapy made metastasis or infection unlikely. Given the lesion’s difficult anatomical access along with the risks of a frontal bone biopsy (e.g., cosmetic defect, potential for infection, proximity to sinus/brain), the absence of progressive neurological deficit, and the likelihood that an additional biopsy would not change management, the multidisciplinary team (neurology, respiratory, rheumatology, and radiology) agreed to treat presumptively. This approach is consistent with international guidelines, which state that once tissue confirmation of sarcoidosis is obtained, additional biopsies are unnecessary if other lesions are radiologically and clinically compatible with sarcoid disease [8].

Management and outcome

The patient was commenced on prednisolone 40 mg daily for four weeks, followed by a structured taper of 5 mg every two weeks until reaching 20 mg, and then 2.5 mg every two weeks until stabilising at 10 mg daily. He remained on 10 mg as long-term maintenance for almost two years due to multisystem sarcoidosis. He demonstrated early clinical improvement in headache and cranial-nerve-related symptoms. Interval CT head imaging approximately two years later showed near-complete healing of the left frontal bone lesion, with no residual or new calvarial abnormalities. CT chest showed stable mediastinal and hilar lymphadenopathy without progression of pulmonary sarcoidosis. Serial pulmonary function tests further confirmed treatment efficacy.

Prior to initiating corticosteroids, spirometry demonstrated reduced lung function with a forced vital capacity (FVC) of 82.4% predicted and forced expiratory volume in one second (FEV₁) of 82.0% predicted. After approximately two years of maintenance prednisolone therapy, spirometry showed improvement to an FVC of 87.3% predicted and FEV₁ of 89.5% predicted, with stable lung volumes and symptom resolution. Based on the stable clinical status, near-complete radiological resolution of the frontal bone lesion, stable thoracic imaging, and improved spirometry, the plan was to taper prednisolone gradually by 2.5 mg per month, with the intention to discontinue therapy entirely.

## Discussion

Osseous sarcoidosis represents an uncommon manifestation of systemic sarcoidosis and can resemble metastatic, infectious, other granulomatous diseases or plasma cell disorder on imaging [1,9]. Skull involvement in sarcoidosis is rare and has been described only in a limited number of published reports [10-12].

Pathophysiology

Granuloma formation occurs through activation of CD4-positive T cells and macrophages against unidentified antigens, resulting in the release of cytokines such as interleukin-2, interferon-γ, and tumour-necrosis-factor-α [13-15]. Persistent immune signalling drives chronic inflammation, fibrosis, and eventual tissue remodelling. Genetic associations such as HLA-DRB1*0301 and HLA-DRB1*1101 have been associated with increased susceptibility [16].

Radiologic and clinical features

Skeletal sarcoidosis can appear lytic, permeative, or sclerotic [17]. Skull or skull-base disease may manifest with localised pain, swelling, or cranial neuropathies. MRI findings are non-specific, so histopathological confirmation remains the diagnostic standard when prior tissue evidence is absent. In this patient, however, the established diagnosis of systemic sarcoidosis and typical radiological appearance allowed us to confidently attribute the frontal bone lesion to sarcoid involvement, avoiding unnecessary invasive biopsy [8].

Serum ACE levels may be elevated but lack specificity, as they may also be elevated in conditions such as tuberculosis or lymphoma [17]. Demonstration of non-caseating granulomas from any representative site, therefore, remains essential for diagnostic confirmation [3,8].

Treatment and prognosis

Systemic corticosteroids are the first-line therapy [18]. For steroid-refractory or relapsing disease, immunosuppressive agents such as methotrexate or azathioprine may be introduced [19]. Early recognition and prompt treatment are vital to prevent irreversible skeletal destruction and neurological complications. Long-term follow-up is essential to monitor for recurrence of skeletal disease or progression of systemic sarcoidosis.

This case underscores that coordinated multidisciplinary management involving neurology, respiratory medicine, rheumatology, and radiology can achieve excellent clinical and radiological outcomes.

## Conclusions

This case highlights an unusual manifestation of sarcoidosis presenting as an aggressive frontal bone lesion that can closely mimic metastatic or malignant bone disease. Recognising this rare form is essential to avoid unnecessary invasive investigations, particularly when systemic sarcoidosis has already been histologically confirmed elsewhere and radiologic findings are typical. A single representative tissue biopsy, combined with concordant clinical and imaging features, is often sufficient for diagnosis and may obviate the need for additional site-specific biopsies. Timely initiation of corticosteroid therapy can result in rapid clinical improvement and radiological remission. This case also underscores the importance of coordinated multidisciplinary care to ensure accurate diagnosis, prevent misinterpretation as malignancy, and achieve favourable patient outcomes.
